# Profile of hepatic involvement in dengue infections in adult Pakistani population

**DOI:** 10.12669/pjms.334.13026

**Published:** 2017

**Authors:** Somia Iqtadar, Nabeel Akbar, Naima Huma, Fawad Ahmad Randhawa

**Affiliations:** 1 Dr. Somia Iqtadar, FCPS (Medicine). Associate Professor of Medicine. King Edward Medical University, Lahore, Pakistan; 2 Dr. Nabeel Akbar, MBBS. Postgraduate Resident of Cardiology. Punjab Institute of Cardiology, Lahore, Pakistan; 3 Dr. Naima Huma, MBBS. Postgraduate Resident of Medicine, King Edward Medical University, Lahore, Pakistan; 4 Dr. Fawad Ahmad Randhawa, FCPS (Medicine). Assistant Professor of Medicine, King Edward Medical University, Lahore, Pakistan

**Keywords:** Alanine aminotransferase, Aspartate aminotransferase, Dengue, Hepatomegaly, Hyperbilirubinemia

## Abstract

**Objectives:**

To estimate the range of hepatic involvement in dengue infections by assessing clinical and biochemical profile of adult dengue infected patients.

**Methods:**

Serologically confirmed 220 adult cases of dengue infections admitted to Mayo hospital from June 2013 to November 2013 were classified as having dengue fever, dengue haemorragic fever and dengue shock syndrome. The frequency and range of bilirubin, liver enzymes derangement and presence of liver enlargement in each group was calculated and further stratified according to age and gender. Patients with positive viral serology, chronic liver disease, malaria and typhoid were excluded from the study.

**Results:**

About 60% of DHF patients had hepatomegaly compared to 40% of DF patients. Liver dysfunction was more common in DF compared to DHF (38.15 vs 18.6%). Hyperbilirubinemia was noted in 40 (18.2%) patients, 28 (12.7%) in DF and 12(5.5%) in DHF. The mean serum bilirubin was higher in DHF [0.87+0.33] compared to DF [0.74+0.27]. Bilirubin was higher in male patients and in younger (<20 years) age group. ALT was elevated more frequently in male patients in age group of 31-40 years and in DF patients as compared to DHF [72(32.7% vs 40(18.2%)]. The mean serum ALT level was 103.7 U/l in DHF and 69.2U/l in DF. AST was raised in all DHF patients as compared to DF in which 40% patients had normal AST levels. Alkaline Phosphate was high in all DHF patients with a mean of 278.7. It was raised in most of the DF patients as well and majority of patients were in age group of 31-40 years.

**Conclusion:**

Liver involvement is very common in dengue infections and is not limited to elevation of transaminases only. Bilirubin and Alkaline phosphatase are also raised in considerable number of patients. Therefore in adults with fever, jaundice, hepatomegaly and altered liver function tests, the diagnosis of dengue infection should be strongly considered in areas where dengue infection is endemic.

***List of abbreviations:***

**DF:** Dengue Fever

**DHF:** Dengue Hemorrhagic Fever

**DSS:** Dengue Shock Syndrome

**DIC:** Disseminated intravascular coagulation

**ALT:** Alanine transaminase

**AST:** Aspartate aminotransferase

## INTRODUCTION

Dengue Fever is a vector-borne tropical infection caused by Dengue virus (DENV). It causes four spectra of disease ranging from; an asymptomatic infection to Dengue fever (DF), Dengue Hemorrhagic Fever (DHF) and Dengue Shock Syndrome (DSS).^1^ It has recently emerged as the fastest growing epidemic of the world.^2^ Every year, 100 million people are reported to suffer from dengue fever in the tropical countries with 250,000 cases of DHF taking 24,000-25,000 lives per year. South East Asia which is famous for tourism bears the bulk of the patient burden.^3^ For the last few years, cases have frequently been reported from Pakistan throughout the year with a peak incidence after the rainy seasons. The situation is made worse by floods that strike the country almost every year. A huge epidemic of Dengue Fever struck Punjab in 2011, with 352 reported deaths and there have been smaller outbreaks eversince.^4^

Patients suffering from dengue commonly have liver involvement although it is a non-hepatotropic virus. This is manifested clinically in the form of hepatomegaly along with mild to moderate transaminitis. Infrequently, severe hepatic dysfunction presenting as fulminant hepatic failure is also reported in dengue infection.^5^-^8^ About 65-97% of dengue patients have derangement of liver biomarker aspartate aminotransferase (AST) and alanine aminotransferase, with maximum rise during the convalescence (days 7–10).^9^,^10^ In areas where dengue is common, it is considered as one of the important causes of acute viral hepatitis.^11^ Liver enzyme derangement is associated with prolonged stay in hospital, development and increased severity of complications and is considered a poor prognostic marker.^12^,^13^

This study was performed to determine the effect of dengue infection on the liver profile in patients with dengue fever (DF) admitted in the Medicine Department of a Tertiary Care Hospital in Pakistan. There is paucity of data in this regard in our country as it is fairly a recent disease in Pakistan. Our research will help elucidate the degree and pattern of liver injury in patients of dengue in our country and will help to identify high risk patients.

## METHODS

This hospital based study was conducted in the Medicine Department, Mayo Hospital, Lahore, from June 2013 to November 2013 (Duration of study 6months). Ethical clearance was taken from Institutional Review Board. Patients presenting in OPD or emergency during the above mentioned period having history of fever ≤ 10 days with two or more of retro orbital pain, headache, arthralgia, myalgia, rash, hemorrhagic manifestations (as evidenced by HESS test)^14^, thrombocytopenia (<100,000/mm3) and leukopenia (<4000/mm3) and further serologically confirmed (either by IgM or NS-1) by enzyme-linked immunosorbent assay (ELISA), of both genders aged more than 13 years were included in this study that consented to use of their clinical data for research purposes on admission (Definite cases of dengue fever). A total of 220 patients conformed to the inclusion criteria. As per hospital protocol, all patients were admitted up to 10^th^ day of fever unless any complication developed that needed an extension of admission. DHF was labeled on the presence of plasma leak manifesting as either change of hematocrit ≥ 20% from baseline or accumulation of fluid as pleural effusion or ascites, documented by ultrasound in serologically confirmed Dengue patients and DSS with rapid and weak pulse and narrow pulse pressure <20 mmHg, or hypotension for age in a patient with DF.^15^ Detailed history was taken from all cases and their thorough clinical examination was performed.

A predesigned, pretested proforma was used to collect the data from all patients. For diagnosis of dengue, dengue IgM capture ELISA, complete blood count including hemoglobin (Hb), platelet count (PLC), total and differential leukocyte count and hematocrit (HCT) were performed. Liver function tests were carried out in all patients that included serum bilirubin, alanine transaminase (ALT), aspartate transaminase (AST), and alkaline phosphatase (ALP) and repeated on alternate days. MP slide, Typhidot IgM ELISA, HbsAg and Anti HCV by ELISA, ultrasound abdomen and thorax were also done. Hepatomegaly was labeled on ultrasound abdomen in patients having liver span of >17cm. Confounders were controlled by excluding those with a history of co-morbid conditions like positive viral hepatitis serology, chronic liver disease, malaria and typhoid fever by performing above mentioned relative investigations. SPSS version 20.0 was used for analysis of the data obtained. Mean and standard deviation was used to present quantitative data like age. Qualitative data like gender distribution, presence of liver enlargement, bilirubin and liver enzymes (ALT, AST and ALP) derangement was presented by using frequencies and percentage tables. Age and gender was stratified in order to control effect modifier to see their effects on outcome variables. After stratification t-test was applied with a p-value of ≤ 0.05 taken as significant.

## RESULTS

**Fig. 1:**
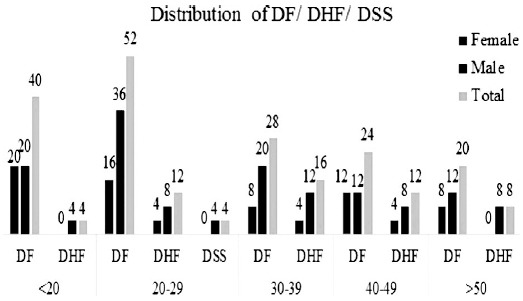
Age and gender distribution of DF, DHF and DSS.

Of the 220 patients included in the study, 164 were diagnosed as DF, 52 as DHF and four as DSS. About 65% of the patients were males and 35% were females. The mean age of the patients was 33.2±9 years but patients having maximum involvement of liver were in the younger age group (20-29 years). All DSS patients were males within 20-29 years’ age group. Details are summarized in Fig. 1. Hepatomegaly was commoner in patients of DHF (60%) as compared to DF (40%) but the difference was not found to be significant statistically. 57% of total patients developed liver dysfunction. Liver functions profile analysis showed that liver dysfunction developed more in DF than in DHF (38.15 vs. 18.6%). Elevated bilirubin levels were noted in 18.2% patients, only 30% of whom had DHF. However, compared to DF (0.74), the mean serum bilirubin was elevated more in DHF (0.87). Bilirubin was higher in young males (<20 years) as compared to older patients. AST was found more commonly elevated as compared to ALT levels (85% vs 51%). ALT had mean value of 69.22, while in DHF it was 103.71. Male patients in age group of 31-40 years and those having DF had more frequent elevations of ALT (32.7% versus 18.2%).

The mean AST found in DF was 68.2 whereas in DHF it was 98.93. AST was raised in all DHF patients as compared to DF, 40% of whom had normal levels. Although, male patients had higher incidence of elevated transaminases but they were found to be more elevated in females. Mean value of AST was 87.3 in females and 73.0 in males. Similarly mean of ALT was found to be 84.1 in females versus 71.7 in males. Elevated levels of alkaline phosphatase were found most in age group of 31-40 years. It was high in all DHF patients (278.7) and in most of the DF patients (mean 264.7). Fulminant hepatitis was not seen in any patient. Details are mentioned in Table-I. In terms of clinical outcome, 92.6% patients were discharged from the hospital and 7.4% expired (p-value 0.011), among which 53.85 % were females (p-value 0.48).

**Table-I:** Comparison of Liver parameters in DF and DHF.

*Disease*	*Test*	*Mean*	*Median*	*SD*	*Range*	*Minimum*	*Maximum*
DF	S. Bilirubin	0.74	0.6	0.27	1	0.4	1.4
	ALT	69.22	54	53.85	208	20	228
	AST	68.24	48	53.35	267	20	287
	Alkaline PO4	264.8	243	101.92	637	113	750
DHF	S. Bilirubin	0.88	0.8	0.34	1.3	0.5	1.8
	ALT	103.71	88	81.27	303	41	344
	AST	98.928	74	69.67	230	50	280
	Alkaline PO4	278.71	230	152.41	553	118	715

## DISCUSSION

In this study, a total of 220 adult patients (ages 14-60 years) fulfilling criteria of dengue fever were enrolled. Liver dysfunction was found to be highest in 20-29 age groups. Other studies done in Pakistan have also shown similar findings. Riaz and Parkash reported the mean age of patients in their studies with dengue were 31±12.9 and 31.87±13.55 years respectively.^3^,^16^ Study done by Hakim has shown that DHF is more prevalent in between 20-45 years of age.^17^ Similarly, study done by Faiz et al. in Faisalabad showed that dengue was more prevalent in 16-30 years’ age group.^18^ 65% of the 220 patients were male and 35% were female. Study done by Mahmood in the same region has shown that there was male predominance (53.8%). Recently, Jehangir and Asar Khan studied the incidence of dengue in KPK and found that their 68.6% patients were males and 31.3% were females.^19^

Similarly, Francisca in Brazil reported 53.25% of the affected patient population was male and 46.75% was female. In Mexico in 1980, Kaplan performed study on epidemiology of dengue fever and reported that dengue infection was more likely to develop in females.^20^ The differences in gender distribution have been explained by different hypothesis as studies that are done in hospitals only depict the patient population who reach out for treatmentand is not a true representation of the actual affected population. Kaplan explained that transmission of disease indoors throughout day affected more females in contrast to males, whereas Goh and Ooi reasoned that as the vector of the disease Aedes is distributed in outdoor environment, it caused lower incidence among females.^21^,^22^

During hospital stay, 57% patients developed liver dysfunction. The frequency of liver involvement was more on DF in contrast to DHF (38.15 vs 18.6%). 51% of the patients showed elevated ALT levels and 85% had elevated AST patients however ALT was mildly more elevated than AST. Souza studied the incidence of hepatitis and aminotransferase changes in patients of dengue in Brazil. In his study acute hepatitis was found only in 3.8% of the patients while 45% patients had altered ALT levels (mean 86U/L) and 63.4% had altered AST levels (93.3%). The contrast in the aminotransferase elevation pattern seen between his study and ours can be due to differences between the baseline levels of these enzymes that are subject to environmental, social and cultural values that include alcoholism, dietary habits and misuse of drugs between the two populations. This study focused only on hospitalized patients while theirs only had outdoor patients.

Ahmed studied the relationship of altered liver enzymes with severity of dengue infection and in his study AST was elevated twice as much as ALT.^23^ Parkash studied the correlation of acute hepatitis with dengue outcome in Karachi, Pakistan and in his study median ALT was 88.5 and AST was 174. AST was reported to be elevated in 95% of the patients while ALT in 86%. These findings can be due to the fact that Parkash study only included patients of DHF, whereas our study included both hospitalized DF and DHF. We can also conclude that this can also be due to secondary infections of viruses with same or different strain.

This study excluded patients having a prior history of liver disease so the effect of hepatitis A, D and E could not be studied. Moreover, exposure to drugs causing liver damage was also not analyzed. The pathogenesis of liver enzymes derangement in patients of dengue is not clear as ALT is only expressed in liver and AST is also found in the red blood cells, heart, skeletal muscle, brain and kidneys.^24^, ^25^ About 55.68% who had “acute hepatitis” were males and 44.32% were females (p-value 0.68). After age stratification, 38% of patients who developed acute hepatitis were aged<25 years, 64.2% were in between 26-45 years and 25% were in 46-60 years age group (p-value 0.698). These findings are also in contrast to Souza’s study that showed frequency of liver damage among females to be 74.6% but the aminotransferase levels were not found to be significantly different from males.

In terms of mortality, our study showed higher rate(7.4%) among patients who developed hepatic involvement as compared to Parkash (2.7%) and Lee (0.1%). The sharp difference in the death rate observed in our patients can be due to difference in the virulence of dengue strains and also to more hepatic damage as a result of repeated infections every year.

### Limitations of the study

The study was carried out in a single tertiary care hospital of Lahore, use of any prior or concomitant hepatotoxic drugs was not ruled out. Also patients with hepatitis A and E could not be excluded as the tests were expensive and are not routinely available in the hospital. Patients suffering from secondary infection were not excluded which can cause bias in the results. The etiology of the liver involvement in this case needs to be elucidated by carrying out research. Other markers of liver damage like prothrombin time, activated partial thromboplastin time, albumin and total proteins should also be looked into and the effect of dengue infections on these and their relation with elevation of transaminases should be studied.

## CONCLUSION

Around 2/3^rd^ of the patients suffering from dengue have liver involvement. Elevation of transaminases may be related to worse outcomes. So such patients should be carefully followed up and managed in order to avoid and pinpoint emergence of complications. Also avoidance of hepatotoxic drugs should be practiced in all febrile patients in areas where dengue is endemic so that liver damage is not further aggravated if the patient has dengue infection.

### Authors’ Contribution

**SI:** Conceived research idea, collected data and manuscript writing.

**NA:** Data collection, analysis and manuscript writing.

**NH:** Data collection, manuscript writing and formatting.

**FAR:** Manuscript writing.
